# (*E*,*E*)-1-(2-Hydroxy­imino-1-phenyl­ethyl­idene)semicarbazide monohydrate

**DOI:** 10.1107/S1600536809013865

**Published:** 2009-04-18

**Authors:** Aslı Öztürk, İlknur Babahan, Nursabah Sarıkavaklı, Tuncer Hökelek

**Affiliations:** a Hacettepe University, Department of Physics, 06800 Beytepe, Ankara, Turkey; b Adnan Menderes University, Department of Chemistry, 09010 Aydın, Turkey

## Abstract

In the title compound, C_9_H_10_N_4_O_2_·H_2_O, the oxime unit has an *E* configuration, and an intra­molecular N—H⋯N hydrogen bond results in the formation of a planar five-membered ring, which is oriented with respect to the aromatic ring at a dihedral angle of 74.82 (17)°. In the crystal structure, inter­molecular O—H⋯O and N—H⋯O hydrogen bonds link the mol­ecules and *R*_2_^2^(8) ring motifs are apparent.

## Related literature

For general background, see: Balsamo *et al.* (1990 ▶); Marsman *et al.* (1999 ▶); Karle *et al.* (1996 ▶); Etter *et al.* (1990 ▶). For related structures, see: Sarıkavaklı *et al.* (2007 ▶, 2008 ▶); Özel Güven *et al.* (2007 ▶); Hökelek, Batı *et al.* (2001 ▶); Hökelek, Zülfikaroğlu *et al.* (2001 ▶); Büyükgüngör *et al.* (2003 ▶); Hökelek *et al.* (2004 ▶); Hökelek *et al.* (2004*a* ▶,*b* ▶). For reference structural data, see: Allen *et al.* (1987 ▶). For ring motifs, see: Bernstein *et al.* (1995 ▶).
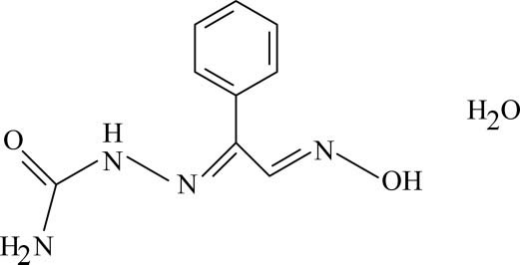


## Experimental

### 

#### Crystal data

C_9_H_10_N_4_O_2_·H_2_O
*M* *_r_* = 224.23
Triclinic, 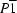

*a* = 5.5593 (2) Å
*b* = 8.2701 (3) Å
*c* = 12.6193 (5) Å
α = 71.900 (3)°
β = 89.998 (5)°
γ = 78.538 (5)°
*V* = 539.29 (4) Å^3^
*Z* = 2
Mo *K*α radiation
μ = 0.11 mm^−1^
*T* = 294 K
0.40 × 0.25 × 0.20 mm

#### Data collection

Enraf–Nonius TurboCAD-4 diffractometer
Absorption correction: ψ scan (North *et al.*, 1968 ▶) *T*_min_ = 0.968, *T*_max_ = 0.978
1953 measured reflections
1752 independent reflections
867 reflections with *I* > 2σ(*I*)
*R*_int_ = 0.048
3 standard reflections frequency: 120 min intensity decay: 1%

#### Refinement

*R*[*F* ^2^ > 2σ(*F* ^2^)] = 0.060
*wR*(*F* ^2^) = 0.185
*S* = 1.05
1752 reflections
171 parameters
5 restraints
H atoms treated by a mixture of independent and constrained refinement
Δρ_max_ = 0.19 e Å^−3^
Δρ_min_ = −0.34 e Å^−3^

### 

Data collection: *CAD-4 EXPRESS* (Enraf–Nonius, 1994 ▶); cell refinement: *CAD-4 EXPRESS*; data reduction: *XCAD4* (Harms & Wocadlo, 1995 ▶); program(s) used to solve structure: *SHELXS97* (Sheldrick, 2008 ▶); program(s) used to refine structure: *SHELXL97* (Sheldrick, 2008 ▶); molecular graphics: *ORTEP-3* (Farrugia, 1997 ▶) and *Mercury* (Macrae *et al.*, 2006 ▶); software used to prepare material for publication: *WinGX* (Farrugia, 1999 ▶).

## Supplementary Material

Crystal structure: contains datablocks I, global. DOI: 10.1107/S1600536809013865/hb2932sup1.cif

Structure factors: contains datablocks I. DOI: 10.1107/S1600536809013865/hb2932Isup2.hkl

Additional supplementary materials:  crystallographic information; 3D view; checkCIF report

## Figures and Tables

**Table 1:** Hydrogen-bond geometry (Å, °)

*D*—H⋯*A*	*D*—H	H⋯*A*	*D*⋯*A*	*D*—H⋯*A*
N3—H3*A*⋯N1	0.88 (3)	2.32 (6)	2.647 (8)	102 (5)
O3—H31⋯O1	0.88 (7)	1.92 (8)	2.776 (9)	164 (8)
O3—H32⋯O3^i^	0.90 (3)	2.17 (7)	2.909 (11)	140 (7)
N2—H22⋯O3^ii^	0.82 (3)	2.10 (4)	2.901 (10)	162 (5)
N3—H3*B*⋯O1^iii^	0.96 (7)	1.96 (6)	2.909 (8)	169 (6)

Symmetry codes: (i) 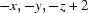; (ii) 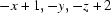; (iii) 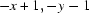, 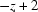.

**Fig. 1.**
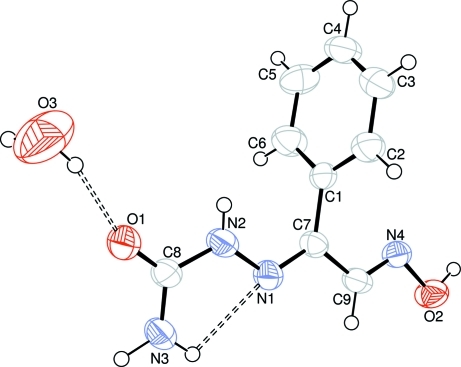
The molecular structure of (I) with displacement ellipsoids for the non-hydrogen atoms drawn at the 50% probability level. Hydrogen bonds are shown as dashed lines.

**Fig. 2.**
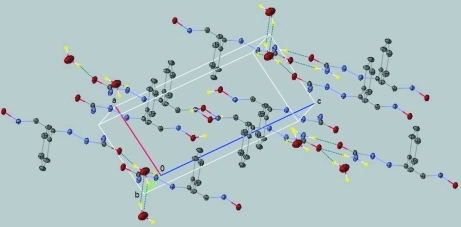
A partial packing diagram of (I). Hydrogen bonds are shown as dotted lines.

## supplementary crystallographic information

### Comment

Oxime and dioxime derivatives have a broad pharmacological activity spectrum, encompassing antibacterial, antidepressant and antifungal activities (e.g. Balsamo *et al.*, 1990).  The oxime (–C=N—OH) moiety is potentially ambidentate, with possibilities of coordination to metal ions through nitrogen and/or oxygen atoms. Oxime groups possess stronger hydrogen-bonding capabilities than alcohols, phenols, and carboxylic acids (Marsman *et al.*, 1999), in which intermolecular hydrogen bonding combines moderate strength and directionality (Karle *et al.*, 1996) in linking molecules to form supramolecular structures; this has received considerable attention with respect to directional noncovalent intermolecular interactions (Etter *et al.*, 1990).

The structures of some oxime and dioxime derivatives have been determined in our laboratory, including those of 2,3-dimethylquinoxaline-dimethyl-glyoxime (1/1), [(II) Hökelek, Batı *et al.*, 2001], 1-(2,6-dimethylphenylamino) propane-1,2-dione dioxime, [(III) (Hökelek, Zülfikaroğlu *et al.*, 2001), *N*-hydroxy-2-oxo-2,*N*'-diphenylacetamidine, [(IV) (Büyükgüngör *et al.*, 2003], *N*-(3,4-dichlorophenyl)-*N*'-hydroxy-2-oxo-2-phenylacetamidine, [(V) Hökelek *et al.*,  2004], *N*-hydroxy-*N*'-(1-naphthyl)-2-phenylacetamidin-2-one [(VI) Hökelek *et al.*, 2004*a*], *N*-(3-chloro-4-methylphenyl)-*N*'-hydroxy-2 -oxo-2-phenylacetamidine [(VII) Hökelek *et al.*, 2004*b*], 2-(1*H*-benzimidazol -1-yl)-1-phenylethanone oxime [(VIII) Özel Güven *et al.*, 2007], (1*Z*,2E)-1-(3,5-dimethyl-1*H*-pyrazole-1-yl)ethane-1,2-dione dioxime [(IX) Sarıkavaklı *et al.*, 2007] and 2-hydroxyimino-1-phenylethanone thiosemicar bazone monohydrate [(*X*) Sarıkavaklı *et al.*, 2008].

As part of our ongoing studies in this area, the structure determination of the title compound, (I), an oxime derivative with one semicarbazide, one phenylacetaldehyde oxime moieties and one uncoordinated water molecule, was carried out in order to investigate the strength of the hydrogen bonding capability of the oxime and semicarbazide groups and to compare the geometry of the oxime moiety with the previously reported ones.

In the molecule of the title compound, (I), (Fig. 1) the bond lengths (Allen *et al.*, 1987) and angles are generally within normal ranges. Ring A (C1—C6) is, of course, planar. The intramolecular N—H···N hydrogen bond (Table 1) results in the formation of a planar five-membered ring B (N1—N3/C8/H3A). The dihedral angle between the planar rings is A/B = 74.82 (17)°.

In the crystal structure, intramolecular O—H···O and N—H···N and intermolecular O—H···O and N—H···O hydrogen bonds (Table 1) link the molecules through *R*_2_^2^(8) ring motifs (Bernstein *et al.*, 1995) (Fig. 2).

### Experimental

Semicarbazide hydrochloride (1.12 g, 10 mmol) and sodium acetate (0.82 g, 10 mmol) were dissolved in double distilled water in the molar ratio 1:1. Then, the solution was mixed with a solution of 2-isonitrosoacetophenone (1.49 g, 10 mmol) in ethanol (10 ml) yielding a turbid mixture. The excess ethanol was added to get a clear solution and was stirred in a magnetic stirrer at room temparature for 4 h. The precipitate formed was filtered, washed with water and dried at room temperature in vacuum desiccator. It was recrystallized from ethanol/water (2:1) solution to yield colourless prisms of (I) (yield; 1.80 g, 85%, m.p. 409 K).

### Refinement

Atoms H9 (for CH), H21 (for OH), H22 (for NH), H3A, H3B (for NH_2_) and H31, H32 (for H_2_O) were located in difference Fourier maps and refined isotropically, with restrains of O3—H31 = 0.88 (7), O3—H32 = 0.90 (3), N2—H22 = 0.82 (3), N3—H3A = 0.88 (3) Å and H31—O3—H32 = 105 (4)° [*U*_iso_(H) = 0.064 (19) Å^2^ (for CH), 0.09 (3) Å^2^ (for OH), 0.040 (17) Å^2^ (for NH), 0.08 (2) Å^2^ (for NH_2_) and 0.125 Å^2^ (for H_2_O)]. The remaining H atoms were positioned geometrically with C—H = 0.93 Å and refined as riding with *U*_iso_(H) = 1.2*U*_eq_(C).

### Crystal data

**Table:**

C_9_H_10_N_4_O_2_·H_2_O	*Z* = 2
*M**_r_* = 224.23	*F*(000) = 236
Triclinic, *P*1	*D*_x_ = 1.381 Mg m^−^^3^
Hall symbol: -P 1	Mo *K*α radiation, λ = 0.71073 Å
*a* = 5.5593 (2) Å	Cell parameters from 25 reflections
*b* = 8.2701 (3) Å	θ = 8.6–17.3°
*c* = 12.6193 (5) Å	µ = 0.11 mm^−^^1^
α = 71.900 (3)°	*T* = 294 K
β = 89.998 (5)°	Prism, colorless
γ = 78.538 (5)°	0.40 × 0.25 × 0.20 mm
*V* = 539.29 (4) Å^3^	

### Data collection

**Table:**

Enraf–Nonius TurboCAD-4 diffractometer	867 reflections with *I* > 2σ(*I*)
Radiation source: fine-focus sealed tube	*R*_int_ = 0.048
graphite	θ_max_ = 24.3°, θ_min_ = 3.4°
Non–profiled ω scans	*h* = −6→0
Absorption correction: ψ scan (North *et al.*, 1968)	*k* = −9→9
*T*_min_ = 0.968, *T*_max_ = 0.978	*l* = −14→14
1953 measured reflections	3 standard reflections every 120 min
1752 independent reflections	intensity decay: 1%

### Refinement

**Table:**

Refinement on *F*^2^	Primary atom site location: structure-invariant direct methods
Least-squares matrix: full	Secondary atom site location: difference Fourier map
*R*[*F*^2^ > 2σ(*F*^2^)] = 0.060	Hydrogen site location: inferred from neighbouring sites
*wR*(*F*^2^) = 0.185	H atoms treated by a mixture of independent and constrained refinement
*S* = 1.05	*w* = 1/[σ^2^(*F*_o_^2^) + (0.0859*P*)^2^] where *P* = (*F*_o_^2^ + 2*F*_c_^2^)/3
1752 reflections	(Δ/σ)_max_ < 0.001
171 parameters	Δρ_max_ = 0.19 e Å^−^^3^
5 restraints	Δρ_min_ = −0.34 e Å^−^^3^

### Special details

**Table:**

Geometry. All e.s.d.'s (except the e.s.d. in the dihedral angle between two l.s. planes) are estimated using the full covariance matrix. The cell e.s.d.'s are taken into account individually in the estimation of e.s.d.'s in distances, angles and torsion angles; correlations between e.s.d.'s in cell parameters are only used when they are defined by crystal symmetry. An approximate (isotropic) treatment of cell e.s.d.'s is used for estimating e.s.d.'s involving l.s. planes.
Refinement. Refinement of *F*^2^ against ALL reflections. The weighted *R*-factor *wR* and goodness of fit *S* are based on *F*^2^, conventional *R*-factors *R* are based on *F*, with *F* set to zero for negative *F*^2^. The threshold expression of *F*^2^ > σ(*F*^2^) is used only for calculating *R*-factors(gt) *etc*. and is not relevant to the choice of reflections for refinement. *R*-factors based on *F*^2^ are statistically about twice as large as those based on *F*, and *R*- factors based on ALL data will be even larger.

### Fractional atomic coordinates and isotropic or equivalent isotropic displacement parameters (Å^2^)

**Table:**

	*x*	*y*	*z*	*U*_iso_*/*U*_eq_	
O1	0.5650 (8)	−0.2847 (5)	0.9749 (4)	0.0635 (13)	
O2	1.6136 (8)	−0.2215 (5)	0.5421 (4)	0.0583 (13)	
H21	1.658 (12)	−0.131 (10)	0.490 (6)	0.09 (3)*	
O3	0.2334 (12)	−0.0741 (10)	1.0657 (7)	0.132 (3)	
H31	0.344 (10)	−0.122 (11)	1.028 (6)	0.125*	
H32	0.088 (7)	−0.084 (10)	1.041 (7)	0.125*	
N1	1.0377 (8)	−0.2812 (6)	0.7948 (4)	0.0456 (13)	
N2	0.8573 (9)	−0.2253 (6)	0.8554 (4)	0.0498 (14)	
H22	0.823 (9)	−0.128 (4)	0.863 (4)	0.040 (17)*	
N3	0.7601 (11)	−0.4938 (7)	0.9070 (5)	0.0594 (16)	
H3A	0.884 (8)	−0.531 (8)	0.872 (5)	0.08 (2)*	
H3B	0.665 (11)	−0.578 (9)	0.942 (5)	0.08 (2)*	
N4	1.4358 (8)	−0.1346 (5)	0.5950 (4)	0.0435 (13)	
C1	1.0910 (10)	0.0218 (7)	0.7132 (5)	0.0389 (14)	
C2	1.2415 (11)	0.0996 (7)	0.7590 (5)	0.0538 (17)	
H2	1.3800	0.0317	0.8044	0.065*	
C3	1.1895 (12)	0.2758 (8)	0.7384 (6)	0.0646 (19)	
H3	1.2905	0.3257	0.7719	0.077*	
C4	0.9938 (13)	0.3786 (8)	0.6700 (6)	0.0602 (18)	
H4	0.9626	0.4987	0.6543	0.072*	
C5	0.8426 (12)	0.3024 (8)	0.6244 (5)	0.065 (2)	
H5	0.7064	0.3716	0.5778	0.078*	
C6	0.8891 (12)	0.1243 (8)	0.6466 (5)	0.0591 (18)	
H6	0.7829	0.0742	0.6162	0.071*	
C7	1.1479 (10)	−0.1688 (6)	0.7314 (4)	0.0387 (14)	
C8	0.7189 (11)	−0.3364 (7)	0.9167 (5)	0.0455 (15)	
C9	1.3383 (11)	−0.2382 (8)	0.6696 (5)	0.0444 (15)	
H9	1.374 (10)	−0.359 (9)	0.687 (5)	0.064 (19)*	

### Atomic displacement parameters (Å^2^)

**Table:**

	*U*^11^	*U*^22^	*U*^33^	*U*^12^	*U*^13^	*U*^23^
O1	0.066 (3)	0.051 (3)	0.078 (3)	−0.018 (2)	0.036 (3)	−0.024 (2)
O2	0.062 (3)	0.044 (3)	0.064 (3)	−0.005 (2)	0.026 (2)	−0.014 (2)
O3	0.106 (5)	0.148 (6)	0.173 (7)	0.002 (5)	0.008 (5)	−0.111 (5)
N1	0.049 (3)	0.041 (3)	0.048 (3)	−0.012 (2)	0.017 (3)	−0.013 (2)
N2	0.058 (3)	0.036 (3)	0.057 (3)	−0.015 (3)	0.019 (3)	−0.014 (3)
N3	0.065 (4)	0.043 (3)	0.076 (4)	−0.022 (3)	0.030 (3)	−0.019 (3)
N4	0.046 (3)	0.037 (3)	0.046 (3)	−0.006 (2)	0.009 (2)	−0.012 (2)
C1	0.038 (3)	0.035 (3)	0.041 (3)	−0.007 (3)	0.011 (3)	−0.010 (3)
C2	0.050 (4)	0.042 (4)	0.066 (4)	−0.005 (3)	−0.008 (3)	−0.014 (3)
C3	0.062 (4)	0.046 (4)	0.089 (5)	−0.016 (4)	−0.003 (4)	−0.024 (4)
C4	0.074 (5)	0.035 (3)	0.071 (5)	−0.013 (4)	0.013 (4)	−0.016 (3)
C5	0.068 (5)	0.045 (4)	0.067 (5)	0.010 (4)	−0.011 (4)	−0.007 (4)
C6	0.060 (4)	0.045 (4)	0.065 (4)	−0.008 (3)	−0.014 (4)	−0.010 (3)
C7	0.043 (3)	0.032 (3)	0.037 (3)	−0.009 (3)	0.002 (3)	−0.005 (3)
C8	0.049 (4)	0.035 (3)	0.046 (4)	−0.009 (3)	0.012 (3)	−0.005 (3)
C9	0.051 (4)	0.031 (3)	0.051 (4)	−0.011 (3)	0.007 (3)	−0.012 (3)

### Geometric parameters (Å, °)

**Table:**

O1—C8	1.226 (6)	C2—H2	0.9300
O2—N4	1.399 (5)	C3—H3	0.9300
O2—H21	0.91 (8)	C4—C3	1.352 (9)
O3—H31	0.88 (7)	C4—C5	1.368 (9)
O3—H32	0.90 (3)	C4—H4	0.9300
N1—C7	1.281 (6)	C5—C6	1.381 (8)
N2—N1	1.357 (6)	C5—H5	0.9300
N2—C8	1.369 (7)	C6—H6	0.9300
N2—H22	0.82 (3)	C7—C1	1.489 (7)
N3—H3A	0.88 (3)	C8—N3	1.320 (7)
N3—H3B	0.96 (7)	C9—N4	1.264 (7)
C1—C2	1.376 (8)	C9—C7	1.447 (7)
C1—C6	1.365 (8)	C9—H9	0.94 (6)
C2—C3	1.368 (8)		
			
N4—O2—H21	101 (4)	C3—C4—C5	118.6 (6)
H31—O3—H32	105 (4)	C3—C4—H4	120.7
C7—N1—N2	118.1 (5)	C5—C4—H4	120.7
N1—N2—C8	120.5 (5)	C4—C5—C6	121.0 (6)
N1—N2—H22	126 (4)	C4—C5—H5	119.5
C8—N2—H22	113 (4)	C6—C5—H5	119.5
C8—N3—H3A	122 (4)	C1—C6—C5	120.0 (6)
C8—N3—H3B	123 (4)	C1—C6—H6	120.0
H3A—N3—H3B	115 (6)	C5—C6—H6	120.0
C9—N4—O2	112.3 (4)	N1—C7—C1	126.4 (5)
C2—C1—C7	121.7 (5)	N1—C7—C9	114.9 (5)
C6—C1—C2	118.6 (5)	C9—C7—C1	118.7 (5)
C6—C1—C7	119.7 (5)	O1—C8—N2	119.1 (5)
C1—C2—H2	119.7	O1—C8—N3	124.4 (6)
C3—C2—C1	120.7 (6)	N3—C8—N2	116.5 (5)
C3—C2—H2	119.7	N4—C9—C7	119.3 (5)
C2—C3—H3	119.5	N4—C9—H9	125 (4)
C4—C3—C2	121.1 (6)	C7—C9—H9	116 (3)
C4—C3—H3	119.5		
			
N2—N1—C7—C1	−2.8 (8)	C5—C4—C3—C2	2.2 (10)
N2—N1—C7—C9	179.9 (5)	C3—C4—C5—C6	−0.6 (10)
C8—N2—N1—C7	174.2 (5)	C4—C5—C6—C1	−1.2 (10)
N1—N2—C8—O1	176.6 (5)	N1—C7—C1—C2	106.3 (7)
N1—N2—C8—N3	−4.4 (8)	N1—C7—C1—C6	−75.8 (8)
C6—C1—C2—C3	0.1 (9)	C9—C7—C1—C2	−76.5 (7)
C7—C1—C2—C3	178.0 (6)	C9—C7—C1—C6	101.4 (6)
C2—C1—C6—C5	1.4 (9)	N4—C9—C7—N1	171.4 (5)
C7—C1—C6—C5	−176.5 (6)	N4—C9—C7—C1	−6.1 (8)
C1—C2—C3—C4	−2.0 (10)	C7—C9—N4—O2	−179.2 (5)

### Hydrogen-bond geometry (Å, °)

**Table:**

*D*—H···*A*	*D*—H	H···*A*	*D*···*A*	*D*—H···*A*
N3—H3A···N1	0.88 (3)	2.32 (6)	2.647 (8)	102 (5)
O3—H31···O1	0.88 (7)	1.92 (8)	2.776 (9)	164 (8)
O3—H32···O3^i^	0.90 (3)	2.17 (7)	2.909 (11)	140 (7)
N2—H22···O3^ii^	0.82 (3)	2.10 (4)	2.901 (10)	162 (5)
N3—H3B···O1^iii^	0.96 (7)	1.96 (6)	2.909 (8)	169 (6)

Symmetry codes: (i) −*x*, −*y*, −*z*+2; (ii) −*x*+1, −*y*, −*z*+2; (iii) −*x*+1, −*y*−1, −*z*+2.
